# Schistosome infection in Senegal is associated with different spatial extents of risk and ecological drivers for *Schistosoma haematobium* and *S. mansoni*

**DOI:** 10.1371/journal.pntd.0009712

**Published:** 2021-09-27

**Authors:** Isabel J. Jones, Susanne H. Sokolow, Andrew J. Chamberlin, Andrea J. Lund, Nicolas Jouanard, Lydie Bandagny, Raphaël Ndione, Simon Senghor, Anne-Marie Schacht, Gilles Riveau, Skylar R. Hopkins, Jason R. Rohr, Justin V. Remais, Kevin D. Lafferty, Armand M. Kuris, Chelsea L. Wood, Giulio De Leo

**Affiliations:** 1 Hopkins Marine Station, Stanford University, Pacific Grove, California, United States of America; 2 Stanford Woods Institute for the Environment, Stanford University, Stanford, California, United States of America; 3 Emmett Interdisciplinary Program in Environment and Resources, Stanford University, Stanford, California, United States of America; 4 Biomedical Research Center EPLS, Saint-Louis, Senegal; 5 Station d’Innovation Aquacole, Saint-Louis, Senegal; 6 Université Lille, CNRS, INSERM, CHU Lille, Institut Pasteur de Lille, U1019-UMR 9017-CIIL-Center for Infection and Immunity of Lille, Lille, France; 7 National Center for Ecological Analysis and Synthesis, Santa Barbara, California, United States of America; 8 Department of Applied Ecology, North Carolina State University, Raleigh, North Carolina, United States of America; 9 Department of Biological Science, University of Notre Dame, Notre Dame, Indiana, United States of America; 10 Division of Environmental Health Sciences, School of Public Health, University of California, Berkeley, Berkeley, California, United States of America; 11 Western Ecological Research Center, United States Geological Survey at Marine Science Institute, University of California, Santa Barbara, California, United States of America; 12 Department of Ecology, Evolution, and Marine Biology, University of California, Santa Barbara, California, United States of America; 13 School of Aquatic and Fishery Sciences, University of Washington, Seattle, Washington, United States of America; Centers for Disease Control and Prevention, UNITED STATES

## Abstract

Schistosome parasites infect more than 200 million people annually, mostly in sub-Saharan Africa, where people may be co-infected with more than one species of the parasite. Infection risk for any single species is determined, in part, by the distribution of its obligate intermediate host snail. As the World Health Organization reprioritizes snail control to reduce the global burden of schistosomiasis, there is renewed importance in knowing when and where to target those efforts, which could vary by schistosome species. This study estimates factors associated with schistosomiasis risk in 16 villages located in the Senegal River Basin, a region hyperendemic for *Schistosoma haematobium* and *S*. *mansoni*. We first analyzed the spatial distributions of the two schistosomes’ intermediate host snails (*Bulinus* spp. and *Biomphalaria pfeifferi*, respectively) at village water access sites. Then, we separately evaluated the relationships between human *S*. *haematobium* and *S*. *mansoni* infections and (i) the area of remotely-sensed snail habitat across spatial extents ranging from 1 to 120 m from shorelines, and (ii) water access site size and shape characteristics. We compared the influence of snail habitat across spatial extents because, while snail sampling is traditionally done near shorelines, we hypothesized that snails further from shore also contribute to infection risk. We found that, controlling for demographic variables, human risk for *S*. *haematobium* infection was positively correlated with snail habitat when snail habitat was measured over a much greater radius from shore (45 m to 120 m) than usual. *S*. *haematobium* risk was also associated with large, open water access sites. However, *S*. *mansoni* infection risk was associated with small, sheltered water access sites, and was not positively correlated with snail habitat at any spatial sampling radius. Our findings highlight the need to consider different ecological and environmental factors driving the transmission of each schistosome species in co-endemic landscapes.

## Author summary

Schistosome parasites infect more than 200 million people worldwide, mainly in sub-Saharan Africa, where many people are at-risk for infection by multiple schistosome species simultaneously. To reduce the global burden of schistosomiasis, control of the parasites’ intermediate host–specific species of freshwater snails–has been elevated in priority to complement mass drug administration campaigns in endemic areas. To maximize the efficacy and efficiency of snail control efforts, a better understanding of where to target intermediate host snails is badly needed. This includes a better understanding of the spatial scale at which snails in the environment contribute to human infection risk, and, in co-endemic settings, how ecological determinants of infection risk vary by schistosome species. We used quantitative snail sampling and remotely-sensed data at 16 villages in the Senegal River Basin to compare and contrast ecological correlates and spatial scales of infection risk from freshwater snails that transmit *Schistosoma haematobium* versus *S*. *mansoni*. We found that infection risk for *S*. *haematobium* was associated with snail habitat at a larger spatial radius than is typically considered for schistosomiasis monitoring and control, whereas infection risk for *S*. *mansoni* was not positively correlated with snail habitat at any spatial sampling radius, but was associated with small water access sites enclosed by emergent vegetation. Our findings highlight the need to consider the different ecological and environmental factors driving the transmission of each schistosome species in co-endemic landscapes.

## Introduction

**Fig 1:**
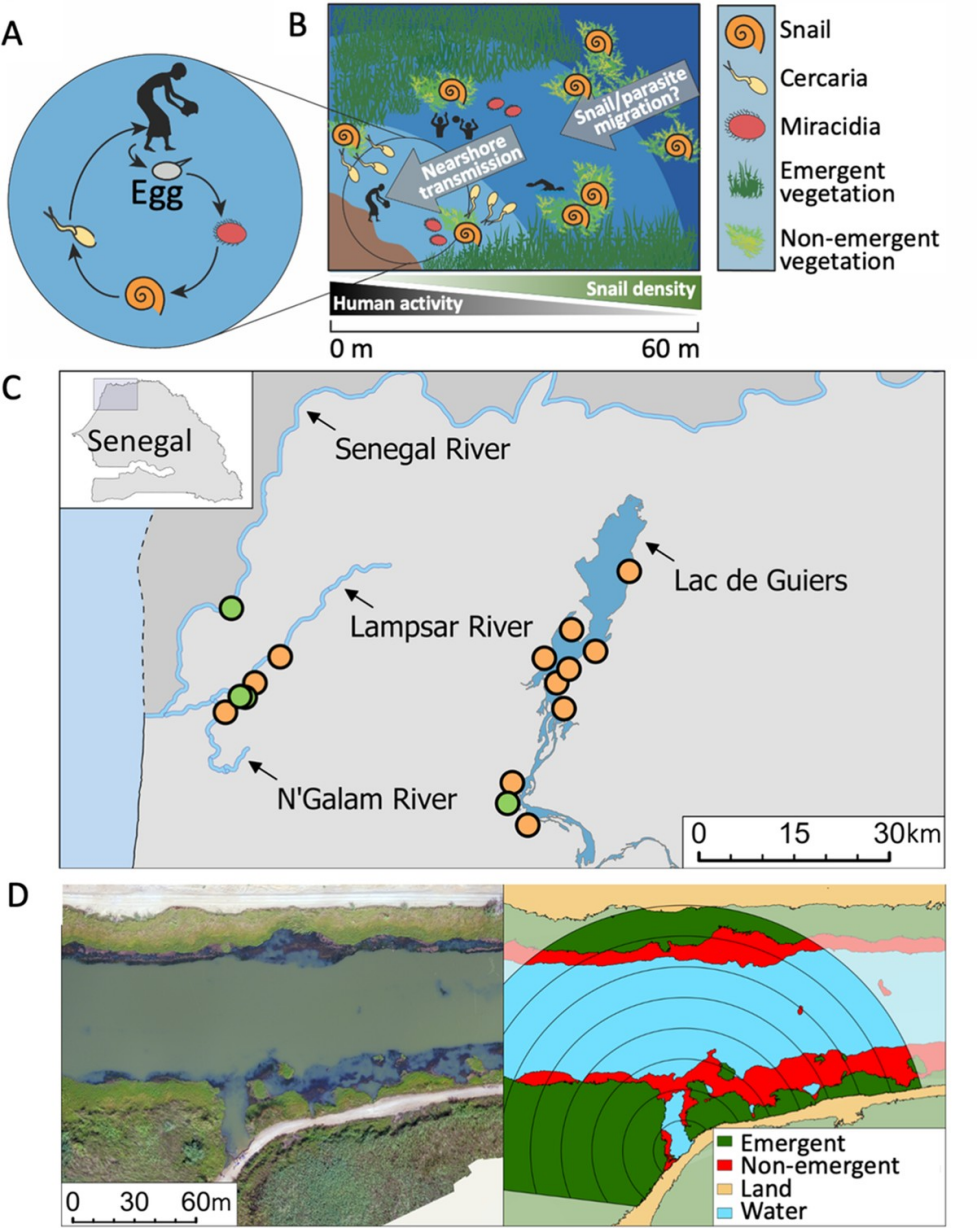
Schistosome life cycle, study design, and study location. A) Schistosome life cycle: eggs are deposited into water by infected humans; miracidia hatch from eggs and infect freshwater snails; larval schistosomes develop and free-swimming cercariae emerge and are infective to humans. B) Visual description of our hypothesized relationship between nearshore human schistosome risk and offshore intermediate host snail populations: we hypothesized that offshore snails represent a population source of susceptible intermediate host snails that, over relatively short periods of time (days), can disperse to nearshore areas and contribute to schistosome transmission cycles. C) Location of 16 study villages (encompassing 32 distinct water access sites) in the Senegal River Basin; 6 villages are located on river settings, and 10 villages are located adjacent to Lac de Guiers. Map was created using ArcGIS Pro v2.8.1 (www.esri.com). D) Overhead drone imagery (left) and classified non-emergent vegetation (red), emergent vegetation (green), water (blue), and land (tan) superimposed by sampling bands used to compare model fit across sampling radii, for a village on a river setting.

*Schistosoma haematobium* and *S*. *mansoni* cause urogenital and intestinal schistosomiasis, respectively, and infect more than 200 million people, mostly in sub-Saharan Africa [1]. Where both schistosomes overlap [2], people can become infected with one or both, depending on which intermediate host snails are present (Fig 1A): *Bulinus* snails transmit *S*. *haematobium* and *Biomphalaria* snails transmit *S*. *mansoni*. Fortunately, a single drug–praziquantel–is highly effective at clearing single and mixed species infections [3,4]. Mass drug administration (MDA) using praziquantel has been the backbone of global schistosomiasis control strategies since 2001 [5]. Unfortunately, MDA does not prevent infection or re-infection from contaminated environments. Persistent transmission in high-burden settings [6–8] highlights the need for complementary environmental control strategies, including snail control [9–13]. Successful snail control requires understanding the ecological conditions that support snail populations and schistosome transmission. This is especially true in co-endemic settings where the ecological conditions that support *S*. *haematobium* and *S*. *mansoni* transmission may differ, and where strategies to identify and target high-risk environments for transmission control may need to be modified accordingly.

Snail control is not a new strategy. It successfully curbed transmission in many countries before praziquantel became widely available, but was subsequently set aside as chemotherapy-based strategies like MDA were embraced [12,14,15]. Alongside a growing body of evidence that snail control can be effective against all major schistosome species [14,16,17], snail control has recently been re-prioritized as a schistosomiasis control strategy to complement MDA [18]. This is underscored by the 2017 World Health Organization (WHO) manual on field use of molluscicides (typically niclosamide-based) in schistosome-endemic areas [19]. But major knowledge gaps remain, especially with regard to how snail biology and ecology affect transmission and control outcomes [15]. For example, the recommended strategy to identify schistosome transmission foci–timed snail searches in the first 10 m distance from the shore of water bodies [19]–yields data that rarely correlate with observed infection outcomes in people [20]. Furthermore, the recommended protocols for applying molluscicides are not tailored to specific snail genera [15], even though different intermediate host species have different life history traits and environmental tolerances [21–25], which could lead to different habitat preferences and spatial distributions within which snails need to be targeted. These gaps limit our ability to effectively and efficiently target snail control strategies, which have associated costs and collateral impacts [19].

This study focuses on better understanding and comparing the relevant sampling design and ecological correlates of transmission risk in the environment for *S*. *haematobium* and *S*. *mansoni*, using a longitudinal cohort study at 16 villages in the lower Senegal River Basin (Fig 1). Here, prevalence for single and mixed schistosome infections among school-aged children is high, despite yearly school-based MDA administered through the National Schistosomiasis Control Program since 1999 [26]. Across study villages, baseline prevalence for *S*. *haematobium* was >77%, *S*. *mansoni* 35%, and mixed infections >30% in 2016. Schistosome transmission intensified in the region after construction of the Diama dam on the lower Senegal River in 1986, before which time *S*. *mansoni* was absent and *S*. *haematobium* transmission was low and seasonal [27,28]. Complex socio-ecological changes have since altered snail abundance, distribution, and parasite compatibility [28,29], creating a landscape of perennial risk for multiple schistosome species.

We suspected that human infection outcomes in the Senegal River Basin may be driven by snail presence further from shore than is typically considered for snail monitoring and control (Fig 1B), and that relevant predictors of risk may differ between the two parasite species. This could arise through spatial variation in water temperature, flow rates, and aquatic vegetation within transmission sites (referred to throughout as ‘water access sites’), which, among other factors, can influence the development, abundance, and distribution of intermediate host snails [30–36]. We also suspected that water access site morphology (i.e., site size and shape features) could differentially influence *Bulinus* and *Biomphalaria* snail abundance [32] and human infection risk for the two parasites, by mediating water access site flow rates, depth, temperature, vegetation, and snail population connectivity within and outside water access sites. Although it is likely that these factors also influence the development, motility, and transmission efficiency of the two larval, free-swimming schistosome stages (miracidia, released from eggs in urine for *S*. *haematobium* and in feces for *S*. *mansoni* to infect snails; cercariae, released by snails to infect humans) [34–40], direct measurement of larval schistosomes–like that for snails–can be labor-intensive, impractical, and imprecise [20,41]. Therefore, we instead attempted to identify indirect and easily measured (i.e., from high-resolution remotely-sensed or drone imagery) indicators of transmission risk in the environment, like snail habitat within a given sampling radius and water access site morphology, that could prove useful for identifying and targeting snail control efforts.

Here, we build upon a recent study by Wood *et al*. [20], which showed that for *S*. *haematobium*, remotely-sensed estimates of snail habitat within water access sites were a more powerful predictor of infection risk in people using those sites than were *Bulinus* spp. snail counts. The Wood *et al*. study counted snails close to shore–within approximately 10 m of shorelines where technicians can safely collect data while wearing personal protective equipment, and consistent with standard infection risk assessment protocols [19]. The study also varied the sampling radius within which they quantified snail habitat, which was up to an order of magnitude larger than the sampling radius used for snail counting. Thus, while they found that snail habitat measured at larger distances from shore than that typically considered in standard risk assessment protocols (i.e., snail counting) may improve *S*. *haematobium* risk prediction, the study did not systematically investigate the optimal radius at which snail habitat correlates with human risk. This raised new questions about how to most effectively measure the relationship between snail populations (via snail habitat) and human infection risk, and whether such strategies should differ for different schistosome–snail species pairs. To address these questions, we first conducted quantitative *Bulinus* and *Biomphalaria* snail sampling within water access sites and up to 200 m outside water access sites, to compare *Bulinus* and *Biomphalaria* snail-habitat associations and spatial distributions. This indicated that both snail species are strongly associated with non-emergent (non-rooted and floating) aquatic vegetation. Then, we systematically measured remotely-sensed snail habitat (non-emergent vegetation) at water access sites within spatial buffers (an arc intersecting the water access site shoreline with a radius extending from a central point on the shore) that ranged from 1 m to 120 m from shorelines. We also measured morphological features of water access sites that could influence local *Bulinus* and *Biomphalaria* abundance (i.e., water access site size and shape characteristics). We used a model comparison approach to determine, if relevant, the optimal sampling radius for correlating snail habitat assessments with infection outcomes in people, as well as the influence of water access site morphology on infection risk, for both *S*. *haematobium* and *S*. *mansoni*.

Our study shows that, in the Senegal River Basin, indirect, remotely-sensed features of water access sites correlated with human infection risk, albeit with different relationships to water access site size and shape characteristics and at different sampling radii between *S*. *haematobium* and *S*. *mansoni*. These findings highlight the need to account for the specific ecological and environmental factors driving transmission of multiple schistosome species in efforts to monitor and target high-risk environments for control in co-endemic settings.

## Methods

### Ethics statement

The study received approval from the National Committee of Ethics for Health Research from the Republic of Senegal (protocol no. SEN14/33) as well as the Institutional Review Boards of Stanford University (protocol no. 32196) and the University of California, Santa Barbara (protocol no. 19–170676). Participants were enrolled in the study only after providing assent alongside written informed consent obtained from their parent or guardian.

### Study village selection and parasitological surveys

Parasitological data collection, ecological field sampling, and unmanned aerial vehicle (drone) imaging [42] took place between 2016 and 2018 at 16 villages in the lower Senegal River Basin, northwestern Senegal (Fig 1C). Data were collected as part of a longitudinal cohort study on regional schistosomiasis transmission ecology and potential for biological control using snail predators [20,43–45]. The criteria for which 16 villages were selected from among 701 candidate villages has been described previously [20]. Briefly, inclusion criteria focused on whether village chiefs reported non-zero *S*. *haematobium* and *S*. *mansoni* prevalence in 2015, whether water access sites were identifiable and heavily used across most seasons, and whether villages were rural. All selected villages are located in close proximity to freshwater and contain between one and four distinct water access sites (Fig 1D) where daily water contact activities take place (e.g., collecting water, bathing, laundry, animal care, swimming, and fishing). Six villages are located on the Senegal River or its tributary, the Lampsar River, and ten villages are located on a large lake fed by the Senegal River, Lac de Guiers (Fig 1C). At the beginning of the study, population sizes ranged from 400 to 1,852 people, and the median population size of villages was 1,000. To eliminate confounding due to a parallel manipulative field experiment, and because limited drone data were available at some locations in one or both years, certain village–time combinations were excluded from the analyses described below (S1 Fig).

Nearly 1,400 school-aged children across 16 selected villages were enrolled in the study after providing assent alongside written informed consent obtained from their parent or guardian. Across all villages, between 31 and 124 children participated in the study (98 children per village on average). In villages where parasitological data were analyzed in this study (S1 Fig), 49.0% of enrolled children were female (578 female, 601 male, 167 unrecorded). In early Spring 2016, all children were screened for baseline *S*. *haematobium* and *S*. *mansoni* infection presence and egg burden according to standard parasitological sampling protocols (described in [46] for *S*. *haematobium* and in [47] for *S*. *mansoni*). A child was considered infected if one or more eggs were present in urine or stool samples, and individual egg burden was measured as the mean egg output across sample replicates. At the village level, egg burden (a proxy for infection intensity) was measured as the geometric mean (Williams mean) egg output among all sampled children [48]; the Williams mean was chosen to deal adequately with zeros and reduce the influence of outliers in over-dispersed data [48,49]. We also measured the prevalence of high-intensity infections, defined as egg burdens exceeding 50 eggs/10mL urine for *S*. *haematobium* or >400 eggs/g feces for *S*. *mansoni*, because egg burdens beyond these cut-offs correspond to the most severe health impacts in people.

At baseline in February-March 2016, infected children were offered treatment with praziquantel (40 mg/kg) to eliminate established schistosome infections. The process was repeated one year later in 2017 and again in 2018, resulting in two 12-month follow-up periods for which annual re-infection (where re-infection is defined as zero or positive infection during the 12 months following praziquantel administration) and egg burden accumulation were recorded for both schistosome species (S2 Fig). All sample collection and processing were conducted by the Biomedical Research Center Espoir Pour La Santé in Saint-Louis, Senegal.

### Field surveys on snail–habitat associations within water access sites

Between May 2016 and February 2018, we conducted seasonal snail sampling at 32 water access sites distributed across the 16 study villages (Fig 1C) to assess snail–habitat associations. Across the 16 villages, six contained one water access site, another six contained two water access sites, two contained three water access sites, and another two contained four water access sites. We conducted snail and environmental sampling three times per year (i.e., per 12-month period during which infection in children was measured), once per each of three dominant climatic seasons in the Sahel ecoregion: warm-dry “spring” (February–May, with mean daily temperature between 21-38C and low humidity); warm-wet “summer” (June–September, mean daily temperature between 27-35C and high humidity); and cool-dry “winter” (October–January temperature meeting 17-36C and low humidity) [50]. After excluding site–time combinations potentially confounded by the parallel manipulative field experiment, we included snail sampling data from 16 villages (32 water access sites) in year one, 14 villages (30 water access sites) in spring of year two, and 11 villages (24 water access sites) in summer and winter of year two (S1 Fig).

In the Senegal River Basin, *S*. *haematobium* (and *S*. *haematobium/S*. *bovis* hybrids [51]) use *Bulinus truncatus* and *B*. *globosus* snails as obligate intermediate hosts, while *S*. *mansoni* uses *Biomphalaria pfeifferi* snails [52]. Our snail field surveys were designed to focus on collection of these species. *B*. *truncatus* and *B*. *globosus* are grouped in our analyses and referred to as ‘*Bulinus* spp.’ because they are morphologically indistinguishable and, while *S*. *haematobium* has historically been associated with *B*. *globosus* in the lower Senegal River, *B*. *truncatus* has become increasingly abundant and is highly compatible with *S*. *haematobium/S*. *bovis* hybrids [51]. This collective term excludes *Bulinus forskalii* and *Bulinus senegalensis* snails, which are rarely found across our study sites and were excluded from analyses.

Snail and environmental sampling protocols are described in detail elsewhere [20] (though described with reference only to *Bulinus* spp. snails, *Biomphalaria pfeifferi* snails were sampled concurrently), and briefly in S1 Appendix. We quantified snail–habitat associations using species-specific snail counts at the quadrat level as outcome variables (considering *Bulinus* spp. and *Biomphalaria pfeifferi* snails in separate models). Because more quadrats had zero snails present than would be expected from a Poisson or negative binomial distribution, we described snail counts using zero-inflated, negative binomial generalized linear mixed effects models (ZINB GLMM) (package *glmmTMB* [53]). In addition to habitat type (emergent, non-emergent, or water/mud)–our main environmental variable of interest–each model included water depth at the quadrat sampling location as a covariate. Because water flow regimes differ considerably between river and lake settings, and because snail–habitat associations may differ for each species accordingly, we included an interaction between village location (river vs. lake) and habitat type. To account for our nested sampling design, each model included a nested random intercept term where sampling period (1 through 6, representing each season–year combination) was nested within water access site ID, which was in turn nested within village ID. Zero-inflation was modeled with a constant probability across sampling points (intercept-only). We used a model comparison approach (Akaike Information Criterion, AIC [54]) to derive this model formulation, after comparing it to models that included season (warm-dry, warm-wet, and cool-dry) within the nested structure, and to models without a zero-inflation term. We performed model residual diagnostics using the DhARMA package in R [55], and checked for spatial autocorrelation in model residuals using Moran’s *I* statistic in R [56]. We computed estimated marginal means for snail counts per quadrat in each habitat type and contrasts between groups using the *emmeans* package in R [57].

### Field surveys on snail abundance nearshore and offshore at village water access sites

**Fig 2:**
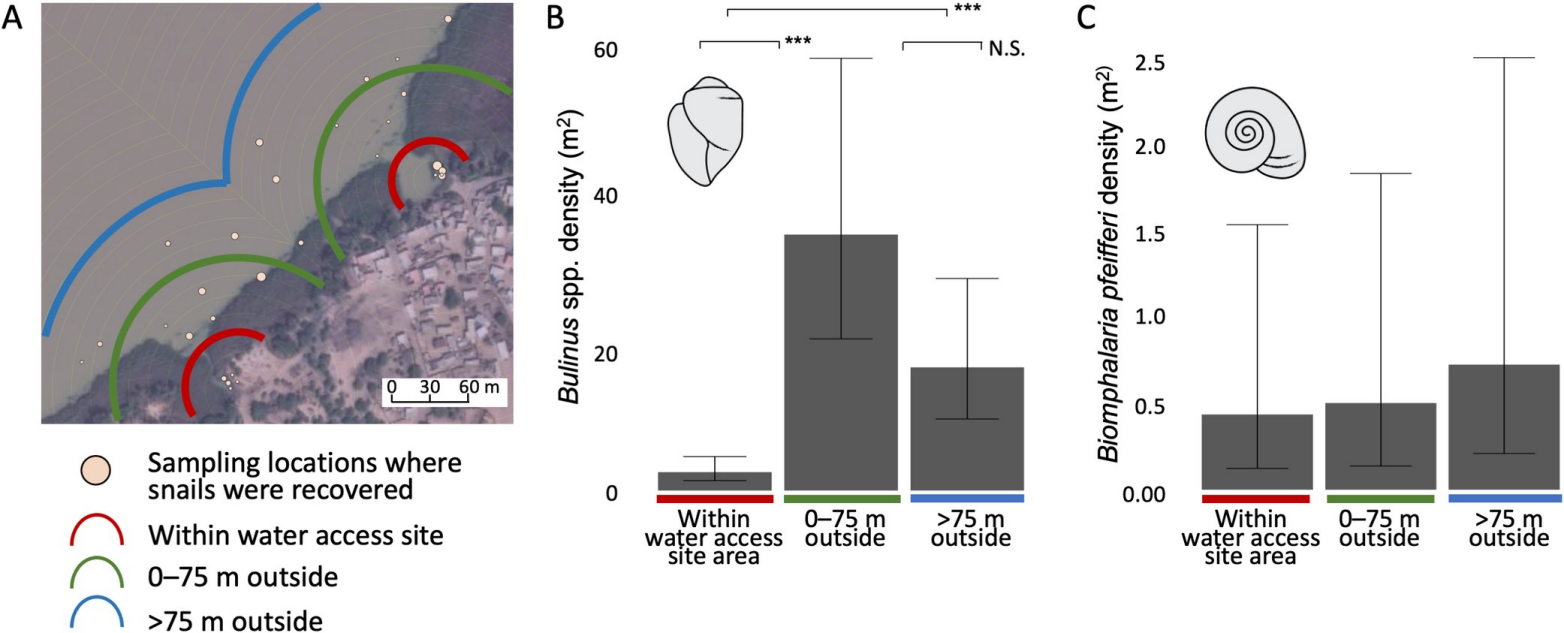
Results from offshore, deep water snail sampling in non-emergent vegetation for three villages located on river settings, and one village located on a lake setting. A) Snails were sampled in three spatial polygons with increasing distances from water access site shorelines: within a discrete water access site (access areas surrounded by emergent vegetation walls, from shorelines to edge of open river channel or lake water), 0 to 75 m outside a water access site area, and 75 m or more outside a water access site area. "Satellite image courtesy of the DigitalGlobe Foundation (now Maxar Technologies, www.maxar.com)." B) *Bulinus* spp. snails were found at their highest density offshore in non-emergent vegetation outside water access sites, and at their lowest density nearshore inside water access sites. C) *Biomphalaria pfeifferi* snails were found in equal densities across the three spatial polygons sampled. For each snail species, estimated marginal means are shown with 95% confidence intervals, and p-values for pairwise comparisons were adjusted for multiple comparisons. *p < 0.05, **p < 0.01, ***p < 0.001.

Quantitative field surveys on snail density and habitat associations described above were logistically restricted to water depths <100 cm, because sampling at deeper depths is unsafe for field technicians. This limited sampling to areas within ~10 m of shorelines, which is consistent with WHO guidelines recommending snail sampling within 10 m of shorelines [19] and with relevant literature suggesting that nearshore habitats pose the greatest risk for transmission to humans [32]. Even so, detailed drone mapping of village water access sites and surrounding aquatic areas revealed extensive snail habitat (defined as non-emergent vegetation, as determined through the snail–habitat association study described above and confirmed in the same study area in [20] and [45]) beyond the 10 meters sampling area from the shoreline (as shown in Fig 2A). We hypothesized that, if non-emergent vegetation beyond nearshore areas supports dense snail populations, such areas might represent a source of unrecognized snail and schistosome exposure risk to humans (Fig 1B). Specifically, we hypothesized that offshore snails (where “offshore” represents areas >10 m from shorelines) might represent a population source of susceptible intermediate host snails that, over time (days), could disperse to nearshore areas and contribute to schistosome transmission cycles. Before testing this hypothesis (via methods described in the next section), we wanted to confirm the presence of intermediate host snails in offshore non-emergent vegetation and compare snail density to that in nearshore areas typically targeted for snail monitoring, human transmission risk assessment, and snail control, as described below.

In July–August 2018, we conducted quantitative offshore snail surveys (i.e., beyond 10 meters from shoreline in water deeper than 1m) at one village on the Senegal River (two water access sites), two villages on the Lampsar River (three water access sites), and one village on Lac de Guiers (one water access site) (S1 Fig). Prior to sampling, we used ArcGIS to generate three sampling polygons of varying distance from water access site shorelines: (i) a nearshore sampling polygon using predefined sampling areas within water access sites, consistent with the snail–habitat association study efforts described in S1 Appendix (water access site edges extended between 10 m and 27 m offshore, but sampling was restricted to < ~10 m from shorelines); (ii) a medium-distance sampling polygon extending 75 m from the outside of the nearshore polygon; and (iii) a far-distance sampling polygon extending up to 40–132 m from the outer edge of the medium polygon. The spatial extents of the far-distance polygons were variable because far-distance polygons needed to be adjusted to avoid spatial overlap when villages contained multiple water access sites (see S1 Appendix). Random points (latitude/longitude) were generated and distributed across each polygon. In the field, we sampled five points (following the numeric order of randomly distributed points) in nearshore areas (within water access sites) using the same protocols described in the previous section. In the medium-distance and far-distance polygons (outside water access sites), we used a boat to sample 10 random points per polygon at each water access site. Snails were collected, labeled, and later identified to species.

For both *Bulinus* spp. and *Biomphalaria pfeifferi* snails, we used negative binomial GLMMs (R package *glmmTMB* [53]) to compare snail density across near-, medium-, and far-distance polygons. Snail count at the quadrat level was used as the outcome variable, and sampling quadrat size (m^2^) was used as an offset, which accounted for the fact that the quadrat used for sampling from a boat was a different size than that used for sampling closer to shore. Each model included a fixed effect variable to control for whether the village was located on a river or lake setting. To account for our nested sampling design, each model included a random intercept term for water access site ID nested within village ID. We performed model residual diagnostics using the DhARMA package in R [55] and computed estimated marginal means for average snail density (per species) in each distance category and contrasts between all groups using the emmeans package in R [57].

### Association between human infection and water access site characteristics and snail habitat within varying sampling radii

**Fig 3:**
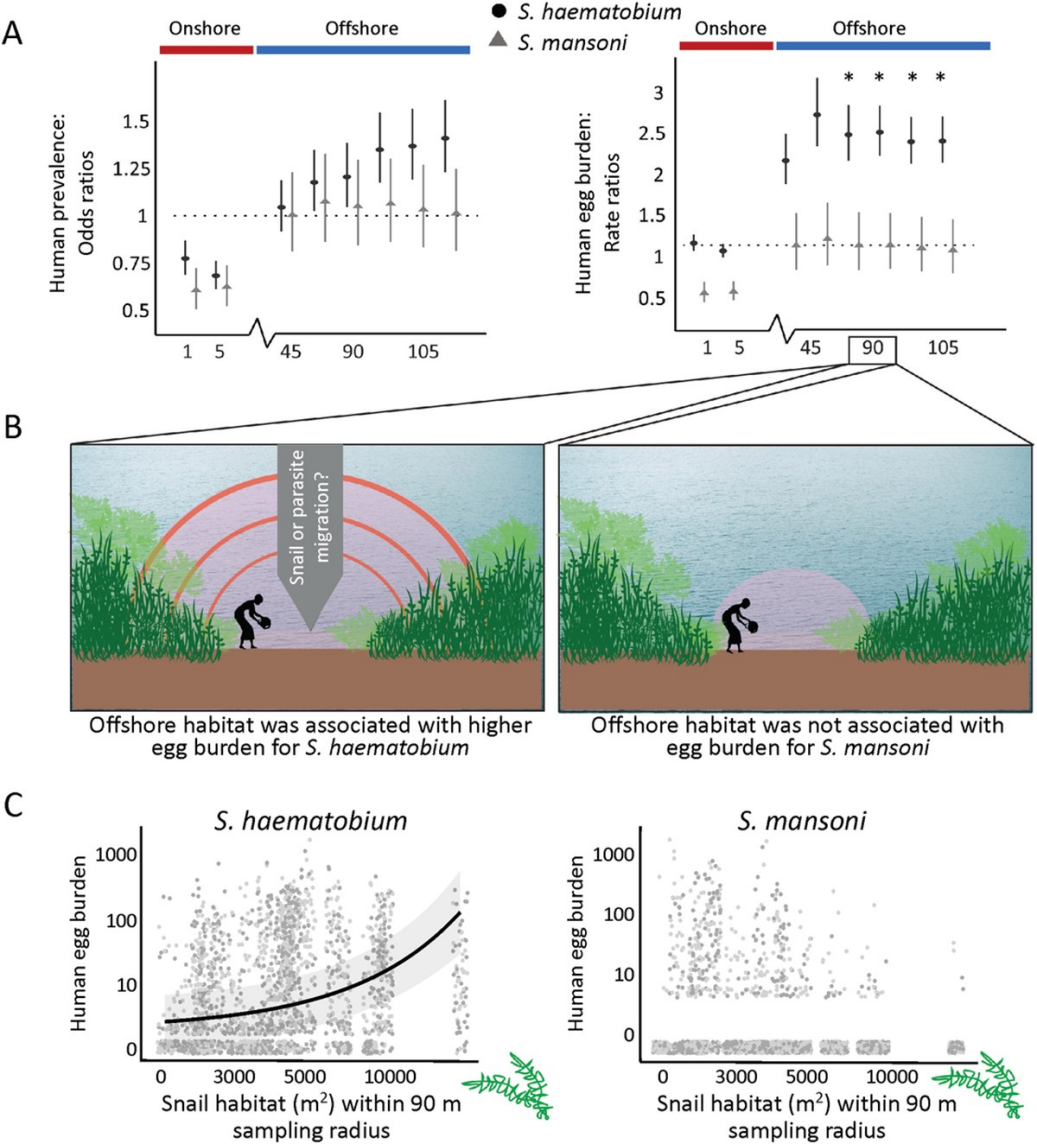
(A) Estimated odds ratios (left) and incident rate ratios (right) reflecting the association between individual infection and egg burden, respectively, for *S*. *haematobium* (black circles) and *S*. *mansoni* (grey triangles) and the total area of non-emergent vegetation measured within different sampling radii; each point estimate and 95% confidence interval were derived from an independent model considering non-emergent vegetation measured at the specified sampling radius (x-axis). Asterisks indicate sampling radii at which a statistical difference (*p < 0.05) between the two species was detected, as determined by an additional model that included an interaction term for species X non-emergent vegetation, independently for each sampling radius. (B) Visual interpretation of results: non-emergent vegetation within a sampling radius of 45 to 90 m was positively associated with *S*. *haematobium* egg burden as compared to *S*. *mansoni*, which was not positively associated with non-emergent vegetation at any sampling radius. (C) Predicted relationship between non-emergent vegetation and *S*. *haematobium* egg burden at a 90 m vegetation sampling radius (95% CI shaded in grey) (left); *S*. *mansoni* (right) was not associated with non-emergent vegetation.

After assessing snail–habitat associations within water access sites and snail density within and outside water access sites, we wanted to determine the optimal sampling radius within which remotely-sensed snail habitat was correlated with schistosome infection outcomes in children. We also wanted to assess whether or not *S*. *haematobium* and *S*. *mansoni* were associated with morphological features of water access sites (i.e., size and shape characteristics). We did this by comparing a set of models that included all site size and shape variables of interest, and that varied only by the sampling radius within which snail habitat was quantified (see S1 Appendix for details on snail habitat mapping using object image analysis). The sampling radii include two “nearshore” spatial extents (1 m and 5 m radius) and six “offshore” spatial extents (45 m, 60 m, 75 m, 90 m, 105 m, or 120 m radii). The site morphological features included in every model were: (1) width of water access site shoreline (m); (2) length of water access site edge (m) (i.e., the linear length of emergent vegetation that form the side “walls” of water access site clearings, from shorelines to river channels or open lake water, Fig 1A); (3) offshore width of water access site openings (i.e., the distance between emergent vegetation site “walls” where they meet the river channel or open lake water); and (4) degree of site ‘circumscription’ (i.e., whether the shoreline width was greater than the offshore width, defining whether a site was partially or fully enclosed by emergent vegetation and thereby disconnected to river channels or open lake water). Site circumscription was included as both a main effect and as an interaction term with village location (river vs. lake). For villages with more than one water access site, circumscription was estimated as a weighted mean of binary (0,1) site-level designations, weighted by water access site size; other site characteristics were estimated as sums across all water access sites. See S1 Appendix for detailed methods on remotely-sensed snail habitat mapping and quantification, and how morphological features were measured for villages with more than one water access site.

We modeled the two parasite species separately, and assessed two relevant outcome variables for each: the probability that a child was re-infected with *S*. *haematobium* or *S*. *mansoni* post-treatment in 2017 and 2018 (binomial GLMMs with logit links, package *lme4* [58]) and individual *S*. *haematobium* or *S*. *mansoni* annual egg burden accumulation (negative binomial GLMMs with log links, package *glmmTMB* [53]). This resulted in comparison of eight models for each response variable, for each species (32 models total). In addition to snail habitat and site morphology, each model controlled for the effect of demographic factors (village population size, child age and sex) on infection outcomes. And, because re-infection in the same children was assessed over time, each model included nested random effect terms for child ID within village ID. Relationships between all model variables were checked to ensure lack of multicollinearity. High-resolution satellite or drone imagery were unavailable in some villages in 2017 and/or 2018. Therefore, we assessed re-infection rates in subsets of children whose water access sites had drone imagery available: 1,171 children across 12 villages in 2016–2017, and 1,349 children across 14 villages in 2017–2018 (S1 Fig).

We used a model comparison approach (AIC) to evaluate model fit within each set of eight models. We then used the best fit models with dAIC <2 [59] to assess the influence of model covariates, to determine (if applicable) the optimal sampling radius at which snail habitat data was correlated to human infection outcomes, and to compare and contrast outcomes for *S*. *haematobium* and *S*. *mansoni*. To further test for differences in infection outcomes between *S*. *haematobium* and *S*. *mansoni*, we repeated our model comparison approach described above but included infection outcomes for both species, and an interaction term for schistosome species and a covariate of interest. This resulted in eight models for each infection presence and egg burden (16 models total), where the models varied only by the sampling radius within which snail habitat was measured. To test for differences in the association of each parasite species with a variable of interest, we included an interaction term for schistosome species by that variable of interest. Covariates of interest were selected as those that suggested differential associations (regression coefficients with opposite signs) with *S*. *haematobium* and *S*. *mansoni* risk or egg burden given results of the individual species models. A significant interaction would suggest a true difference in that variable’s association with village-level infection risk for the two different parasite species. Interaction slopes and contrasts were estimated using the emmeans package in R [57].

## Results

### Parasitological surveys in study villages

Both *S*. *haematobium* and *S*. *mansoni* prevalence and egg burden were high at these study villages. At baseline, *S*. *haematobium* prevalence (mean 77.1%, range 42.5% to 98.8%) and egg burden (8.93 eggs/10ml urine, range 0.71 eggs/10ml to 29.8 eggs/10ml) in school-aged children across all villages was higher than *S*. *mansoni* prevalence (mean 34.9%, range 3.2% to 92.5%) and egg burden (3.38 eggs/g feces, range 0.16 eggs/g to 156 eggs/g) (S1 Table). Drug treatment in 2016 led to substantial reductions in high-intensity infections for both parasite species (25.6% to 0.0% for *S*. *haematobium*, 5.2% to 0.0% for *S*. *mansoni*) by 2017. Re-infection rates and egg burden for both species were nonetheless high in 2017 and 2018: re-infection prevalence for *S*. *haematobium* was 66.3% (range 16.0% to 96.0%) in 2017 and 68.1% (range 31.1% to 98.3%) in 2018, and re-infection prevalence for *S*. *mansoni* was 13.8% (range 0.0% to 51.9%) in 2017 and 25.0% (range 4.1% to 94.4%) in 2018 (S1 Table). Re-infection prevalence for co-infections was 11.9% (range 16.0% to 99.1%) in 2017 and 19.9% (range 0.0% to 51.9%) in 2018 (S1 Table).

### Field surveys on snail–habitat associations within water access sites

*Bulinus* and *Biomphalaria* snails exhibited similar habitat associations, but the strength of the associations differed based on whether water access sites were located in a river or lake setting. In general, more snails of both species were found in water access sites located on the river, and *Bulinus* spp. snails were substantially more abundant than *Biomphalaria pfeifferi* across all sites (Table 1 and S3 Fig).

**Table 1:** Regression table for snail-habitat models (ZINB GLMM) of snail density (snail counts per sampling quadrat) in water access sites as a function of freshwater habitat type, water depth, and village location (lake vs. river), for both *Bulinus* spp. snails and *Biomphalaria pfeifferi*. Snails of both species were most abundant in non-emergent vegetation. In all habitat types, snails were less abundant in village water access sites on lake settings than those on river settings. The number of quadrats sampled differs due to missing data for *Bulinus* spp. snail counts in two quadrats. Coefficients for differences in snail density according to habitat type are shown for non-emergent vegetation and open water with emergent vegetation as the reference category. Model coefficients are shows with standard errors in parentheses. Bolded coefficients represent statistically significant factors. *p < 0.05, **p < 0.01, ***p < 0.001.

	*Bulinus* spp.	*Biomphalaria pfeifferi*
Intercept	0.419 (0.685)	-1.299 (0.930)
Non-emergent vegetation (reference: emergent vegetation)	1.960*** (0.313)	1.361*** (0.397)
Open water (reference: emergent vegetation)	-0.883** (0.288)	-1.117** (0.399)
Location: Lake (reference: River)	-1.816* (0.869)	-3.929** (1.453)
Depth (m, scaled)	0.469*** (0.083)	0.194 (0.119)
Interaction: Non-emergent vegetation X Lake	-1.327*** (0.396)	-1.155* (0.585)
Interaction: Open water X Lake	-1.449*** (0.396)	-1.433 (0.735)
N quadrats sampled	2317	2319
logLik	-2069.159	-741.729
AIC	4162.319	1507.458

*** p<0.001

**p<0.01

*p< 0.05

Considering habitat type, pairwise contrasts of the estimated marginal means for snail counts in each habitat type showed that both *Bulinus* spp. and *Biomphalaria pfeifferi* were found at their highest densities in non-emergent vegetation, followed by quadrats including a mix of non-emergent and emergent vegetation. This is consistent with previously reported snail–habitat associations across the same study sites in [20] and [45]. The exception to this trend was that *Biomphalaria pfeifferi* snail density did not differ between non-emergent vegetation and emergent vegetation along the edges of water access sites on the lake (pairwise contrast of estimated marginal means: p = 0.626). In other words, for *Biomphalaria pfeifferi* at lake sites (where *S*. *mansoni* prevalence in schoolchildren is higher than at river sites), snails were equally likely to be found in non-emergent vegetation and along the edges of dominant emergent reeds that form the dense borders of water access sites and where non-emergent vegetation tends to settle. However, because *Biomphalaria* snails are much rarer than *Bulinus* snails, it is possible that we did not detect a difference between these habitat types due to low statistical power. Both species were absent or only occasionally found in mud-bottom, open water habitats (Table 1 and S3 Fig).

We found that *Bulinus* spp. snail density increased as water depth increased within water access sites (zero-inflated negative binomial GLMMs, Estimate = 0.469, SE = 0.083, p<0.001, Table 1). Because water depth is correlated with distance from shoreline (sampling locations in deeper water are farther from shorelines), this suggests that *Bulinus* spp. snail density increases with distance from shorelines. We found no effect of water depth on *Biomphalaria pfeiffer*i density.

### Field surveys on snail density nearshore and offshore at water access sites

We found dense aggregations of snails in offshore, deep-water habits (Fig 2). The densest aggregations of *Bulinus* spp. snails were in in non-emergent vegetation outside water access sites (Fig 2). Specifically, *Bulinus* spp. snails were at their highest densities in the sampling area extending from the outer edges of water access sites up to 75 m beyond (the “medium” distance sampled), then in non-emergent vegetation in the sampling polygon even farther from the edges of water access sites in open river or lake areas (the farthest distance sampled) (Fig 2A). The lowest densities of *Bulinus* spp. snails were found in non-emergent vegetation within water access sites. In contrast, *Biomphalaria pfeifferi* snail density did not vary across sampling distances (Fig 2B).

### Association between human infection and water access site characteristics and snail habitat within varying sampling radii

#### *S. haematobium* infection probability and egg burden

Nearshore snail habitat (the amount of non-emergent vegetation within 1 m and 5 m sampling radii) was negatively associated with *S*. *haematobium* infection presence in children (Fig 3A and S2 Table). However, the association was positive when snail habitat was measured within a sampling radius of at least 90 m from shorelines (Fig 3A–3C and S2 Table). Among models assessing offshore snail habitat (sampling radii of 45 m to 120 m from shorelines), AIC was lowest for the model fit to snail habitat data measured within a sampling radius of 120 m, but among offshore models, dAIC <2 for models with sampling radii of 90 m to 120 m (S2 Table). Nearshore snail habitat was not associated with *S*. *haematobium* egg burden in children, but snail habitat measured within all offshore sampling radii was (Fig 3A and S3 Table). Across all egg burden models, AIC was lowest for the model integrating snail habitat at a 90 m sampling radius (negative binomial GLMM, Incident Rate Ratio = 2.21, 95% CI = 1.74–2.80, p < 0.001; Fig 3A and S2 Table).

**Fig 4:**
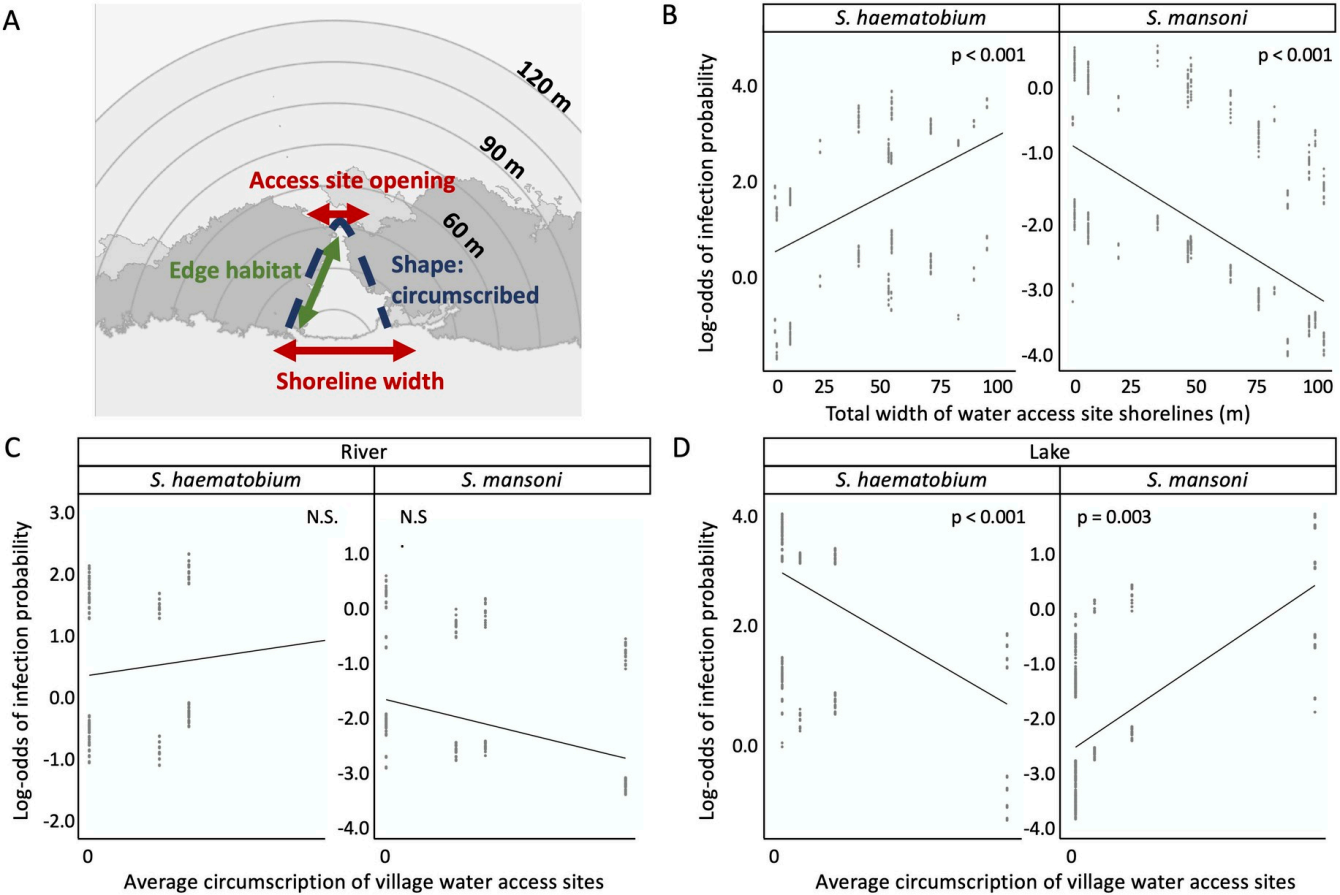
(A) Visual description of size and shape characteristics measured at each water access site; shown is a fully circumscribed water access site, where the man-made site is fully enclosed by emergent vegetation. (B) Prediction interval (solid line) and partial model residuals of a logistic GLMM testing for differences in infection probability for *S*. *haematobium* (left) versus *S*. *mansoni* (right), given the width of water access sites, using an interaction term for effect X schistosome species. (C) Prediction interval and partial model residuals for the effect of circumscription at villages on river settings, assessed using a three-way interaction term for circumscription X schistosome species X location (river versus lake). (D) Prediction interval and partial model residuals for the effect of circumscription at villages on lake settings. For all (B)-(D), pairwise contrasts were derived from a model fit using non-emergent vegetation data measured within a sampling radius of 90 m. All predictor variables were centered and scaled. Full model results are shown in S6 Table.

Water access site size and shape characteristics (Fig 4A) were also associated with *S*. *haematobium* outcomes in children. For infection presence models, water access site shoreline width (Fig 4B), and site edge length were positively correlated with *S*. *haematobium* presence across all models with variable sampling radii for snail habitat (S2 Table). The width of water access site openings (where site edges meet the river channel or open lake water) was negatively correlated with infection presence only in offshore vegetation models (90 to 120 m sampling radii) (S2 Table). Across models, site circumscription differentially associated with re-infection in villages located on a lake vs. river setting (as indicated by a significant interaction between site circumscription and village location) (S2 Table). Site circumscription had no effect on re-infection in villages located on a river (Fig 4C), but was negatively correlated with re-infection in villages located on the lake (i.e., circumscribed sites, or sites with little to no connectivity to open lake water, had lower re-infection rates than open sites) (Fig 4C). For the egg burden model with the lowest AIC (90 m snail habitat sampling radius), the length of water access site edges was the only size and shape characteristic that correlated with *S*. *haematobium* egg burden accumulation in children (90 m negative binomial GLMM, Incidence Rate Ratio = 2.64, 95% CI = 1.04–6.70, p = 0.041; S3 Table).

Across all infection presence models, villages located on a lake setting had higher re-infection rates than those located on a river setting; boys were more likely to be infected with *S*. *haematobium* than girls; and villages with smaller population sizes had higher re-infection rates than villages with larger populations (S2 Table). For all egg burden models, males had higher *S*. *haematobium* egg burdens, and younger children had higher egg burdens than older children (S3 Table).

#### *S*. *mansoni* infection probability and egg burden

For *S*. *mansoni* infection outcomes, snail habitat (non-emergent vegetation) was correlated to neither infection presence nor egg burden accumulation in children at any offshore spatial extent (45 m to 120 m sampling radii) (Fig 3A–3C). Like for *S*. *haematobium*, snail habitat in nearshore spatial extents (1 m and 5 m sampling radius) was negatively correlated to *S*. *mansoni* re-infection rates and egg burdens (S4 and S5 Tables).

Across all infection presence models, water access site shoreline width was negatively correlated with *S*. *mansoni* re-infection (Fig 4B and S4 Table), suggesting that villages with smaller water access sites had higher infection rates than villages with larger water access sites. In the models within dAIC <2 of the top model (1 m and 5 m sampling radius), the length of water access site edges was positively correlated with *S*. *mansoni* re-infection (S4 Table), but this association was not present for offshore vegetation models (45 m to 120 m sampling radii). Across all models with variable snail habitat sampling radii, there was a significant interaction between circumscription and village location on river vs. lake settings, but with opposite outcomes than that for *S*. *haematobium* on lake settings: circumscription had no effect on *S*. *mansoni* re-infection on the river, but was positively correlated with re-infection in villages located on the lake (Fig 4C and 4D and S4 Table). Finally, boys were more likely to be re-infected with *S*. *mansoni* than girls across all models.

Water access site shoreline width was negatively correlated with *S*. *mansoni* egg burden accumulation in all models except for the 1 m snail habitat sampling radius (S5 Table). For all models, circumscription was positively correlated with egg burden accumulation on villages located on the lake, but not on the river (S5 Table). For nearshore vegetation extent models (1 m and 5 m sampling radius), snail habitat was negatively associated with egg burdens, and the length of water access site edges was positively correlated with egg burdens (S5 Table). However, these associations were not present in offshore vegetation models (45 m to 120 m sampling radii). Finally, like that for *S*. *haematobium*, boys had higher *S*. *mansoni* egg burdens than girls across all egg burden models.

#### Interaction model assessing differential snail habitat associations with *S*. *haematobium* and *S*. *mansoni*

All infection presence and egg burden interaction models indicated that there were significant differences between *S*. *haematobium* and *S*. *mansoni* infection outcomes when considering water access site shoreline width and circumscription on river vs. lake settings (S6 and S7 Tables). For snail habitat associations, a differential association between parasite species and snail habitat was not detected for infection presence models at any snail habitat sampling radius (S6 and S7 Tables). However, egg burden models indicated that there was a significant difference between egg burden of the two parasite species given snail habitat measured within sampling radii between 75 m and 120 m (Fig 3A and S6 and S7 Tables).

## Discussion

Our results show that, in the Senegal River Basin, a region co-endemic for *S*. *haematobium* and *S*. *mansoni*, ecological correlates of infection risk differ between the two parasite species. We also show that, for *S*. *haematobium*, human infection risk was positively correlated with remotely-sensed *Bulinus* spp. snail habitat measured within a sampling radius of 90 m or more from water access site shorelines. Similarly, annual *S*. *haematobium* egg burden–which represents cumulative infections over the preceding year–was positively correlated with snail habitat across sampling radii of 45 m or more, with model fit optimal at a 90 m sampling radius. This represents a much larger area than is typically considered relevant for snail monitoring and control activities [19]. In contrast, *S*. *mansoni* infection risk and egg burden was not correlated with snail habitat at these offshore sampling radii (45 m or more). Instead, *S*. *mansoni* was correlated with size and shape characteristics of water access sites such that smaller, more circumscribed sites were associated with the greatest re-infection risk. Our findings yield two potential implications for schistosomiasis risk assessment and environmental control: (i) ecological characteristics of transmission sites might prove useful to predict where schistosomiasis transmission risk is high, but with different associations and at different spatial extents for different schistosome–snail species pairs; and (ii) environmental control strategies, including snail control, may be most effective if tailored to the ecology of targeted schistosome–snail species pairs.

The correlation between the local availability of snail habitat within 90 m or more from shorelines and village-level *S*. *haematobium* infection presence and egg burden could be driven by two general factors. First, a larger radius results in a larger sampling effort, which likely reduces error in snail habitat assessment, especially where snail habitat is heterogeneous. Smaller error should make it easier to observe an association between snail habitat and infection risk. Second, a large sampling radius makes it possible to include habitat farther from shore that might support snails that contribute to infection. This explanation was supported by findings from our snail sampling efforts. Within discrete water access sites–which range in size from about 10 m to 100 m from shorelines–*Bulinus* spp. snail density increased with sampling distance from shorelines, but was highest in snail habitat found outside of water access sites. This suggests that across the dynamic aquatic landscapes in river and lake settings adjacent to villages in the Senegal River Basin, snail habitat measured within a sampling radius of at least 90 m best represents the spatial area within which *Bulinus* spp. snails can–over time–contribute to *S*. *haematobium* infection risk in people close to shore. And, while we did not assess the distribution of infected snails here, a study in a river setting in Zimbabwe showed that schistosome prevalence in *Bulinus globosus* and *Biomphalaria pfeifferi* snails was highest within about 60 m from shorelines [34].

It remains unclear how snails and larval trematodes are moving between nearshore and offshore snail habitats. Regarding snail mobility, mark–recapture studies in low-flow stream habitats in Tanzania [60] and in stagnant pools along a river in Zimbabwe [33] show that *Bulinus globosus* snails can drift tens to hundreds of meters within and between sites in a matter of days, especially following rainfall. Similarly, in Ghana’s Lake Volta [32], it was observed that *Bulinus truncatus rholfsi* can drift into water access sites when attached to vegetation dislodged from deep-water belts of *Ceratophyllum* (the dominant snail habitat in our study sites [20,45]). This supports the hypothesis that offshore snails can contribute to infection dynamics in people at shorelines, where most human water-contact occurs. Regarding larval trematode mobility, passive transport of trematode cercariae across long distances via wind-driven water currents [61] may facilitate transmission between infected snails in offshore environments and humans interacting with water in nearshore environments. In our study system, *Bulinus* spp. snails in particular were found at their highest density in offshore snail habitat. Taken together with our finding that *S*. *haematobium* infection risk is best correlated with snail habitat measured within a sampling radius of 90 m from shorelines, such offshore snail populations may be an under-appreciated source of long-term *S*. *haematobium* disease risk. If so, environmental interventions–like snail control–should be conducted at a radius that matches this spatial area of risk. Alternatively, increasing the temporal frequency of interventions in nearshore areas where transmission typically occurs could reduce transmission risk from a broader spatial area of influence, while also mitigating potential collateral impacts across large scales.

*S*. *mansoni* infection risk, on the other hand, was not associated with the local availability of snail habitat measured within any offshore sampling radius (45 m or larger). This is despite the fact that *Biomphalaria* snails, like *Bulinus*, tend to prefer vegetated habitat over non-vegetated, or mud-bottom habitat (S3 Fig), as has been found elsewhere [62]. In general, however, *Biomphalaria pfeifferi* snails were far less abundant than *Bulinus* spp. snails in this region. In other settings, they have been observed to show strong preference for shallow, low-flow waters [63], and to be less tolerant to high water flow than *Bulinus* snails [62]. Thus, it is possible that in our study area, *S*. *mansoni* infection risk is more strongly associated with water access site features that ensure low flow habitat where *Biomphalaria* snails can survive than with the total amount of non-emergent vegetation locally available to colonize. Future efforts to simulate variation in water flow within and across water access sites–a non-trivial activity that requires collaboration with hydrological modelers–could help inform these findings. At the same time, other unmeasured factors like water temperature and human behaviors may also be important determinants of *S*. *mansoni* risk, and should be incorporated into future field studies.

Surprisingly, both *S*. *haematobium* and *S*. *mansoni* risk were negatively correlated with non-emergent vegetation very close to shorelines (1 m and 5 m sampling radius), where snail monitoring and control activities typically occur [19]. Snail abundance may be depressed in nearshore vegetation as compared to offshore vegetation due to disturbance, where nearshore aquatic plants are constantly trampled by human and animal activity. Furthermore, soap used for bathing and washing can kill snails, cercaria, and miracidia [64]. It is also likely that in areas where nearshore vegetation is less dense, the water access site may also be more heavily used (and therefore contamination and exposure is higher where there is less nearshore vegetation), which could explain one mechanism by which low vegetation very near shore correlates to more schistosome infection in people using those water access sites. In water access sites with more aquatic vegetation, it is possible that vegetation mechanically blocks larval schistosome movement, a phenomenon that has been observed to impact transmission of several helminth species [38]. For example, *Ceratophyllum demersum*, commonly found at our sites, has been found to interfere with *S*. *mansoni* cercarial host-finding, with the potential to reduce downstream infection [65]. This seeming contradiction–that non-emergent vegetation is strongly and positively associated with snails and with *S*. *haematobium* re-infection in children when measured with a large radius, but that non-emergent vegetation very nearshore is negatively associated with re-infection in children–may in fact represent co-occurring mechanisms by which floating vegetation acts to influence schistosome transmission risk differently at different distances from shore. In general, however, it is likely that the positive effect of vegetation on snail abundance at a large radius greatly outweighs the negative effect of vegetation on host-finding behavior close to shorelines.

Size and shape characteristics of water access sites influenced transmission risk for both species, but in different ways. The width of water access site shorelines, which we used as a proxy for water access site size, was positively associated with *S*. *haematobium* infection risk (as previously found in [20]), but shoreline width was negatively associated with *S*. *mansoni* risk. On lake settings, circumscription (the degree by which emergent vegetation enclosed sites from river channels or open lake water) was negatively associated with *S*. *haematobium*, but positively associated with *S*. *mansoni* risk. These different associations, in settings where both snail and parasite species are present, highlight the different ecological aspects of these two species’ transmission. It may be that large and–on the lake–open sites promote *Bulinus* spp. dispersal from snail habitat outside water access sites where *Bulinus* density is highest, to snail habitat nearer to shorelines, where transmission between people and snails is most likely to occur. In contrast, for *S*. *mansoni*, small and–on the lake–enclosed sites may reduce water flow and better support *Biomphalaria pfeifferi* snail populations than large and open sites.

There are several important caveats to our findings. First, variation in socioeconomic and behavioral patterns among villages (i.e., access to sanitation facilities and clean water, outdoor urination versus defecation behaviors, livelihood, education, and water-contact behaviors) can greatly influence risk [64,66–68], and those variables are not measured here. In the future, these factors should be measured alongside ecological and environmental factors related to intermediate host snail distribution for improved transmission risk mapping. Second, lower abundance of both *Biomphalaria pfeifferi* snails in water access sites and *S*. *mansoni* parasites in people, as compared to *Bulinus* snails and *S*. *haematobium*, could have limited our ability to detect statistical associations related to *Biomphalaria* distribution in water access sites and *S*. *mansoni* transmission risk in people. Third, the diagnostic sensitivity to detect low-intensity *S*. *mansoni* infections in people is lower than that for *S*. *haematobium*, which could also have affected our ability to detect statistical associations for *S*. *mansoni* [69]. Fourth, our analyses group *B*. *globosus* and *B*. *truncatus* snails as compatible *S*. *haematobium* hosts; extensive genetic sequencing of the snails we collected could reveal different spatial distributions and schistosome compatibility between the two *Bulinus* species, with potential implications for environmental surveillance and intervention. Finally, this study took place in a limited geographical area. Although the Senegal River Basin is one of the most hyper-endemic regions for schistosomiasis transmission in the world, it is possible that in other geographical settings, snail abundance and schistosome transmission risk are influenced by different ecological drivers.

Through this study, we show that in the Senegal River Basin, spatial extents and ecological correlates of schistosome infection risk differ between *S*. *haematobium* and *S*. *mansoni* in water access sites where their intermediate host snails overlap. The ecological insights we provide may help inform risk monitoring and transmission control activities. For example, *S*. *haematobium* risk assessment in the Senegal River Basin and other areas with similar transmission settings could involve snail habitat mapping within spatial sampling radius of around 90 m from water access site shorelines, while *S*. *mansoni* risk assessment could be focused at small, low-flow, more circumscribed water access sites where snail habitat is protected from strong water currents. These assessments, in turn, can provide guidance on where and at what spatial scale environmental interventions, like snail control, will be most effective for each schistosome species.

Ideally, transmission control strategies in regions co-endemic for *S*. *haematobium* and *S*. *mansoni* would be chosen so that they can effectively target both snail and parasite species simultaneously. Targeted use of chemical molluscicides to control all relevant intermediate host snails in high-risk areas, as the WHO recommends [19], could be effective at a spatial scale or temporal frequency that adequately reduces snail and parasite abundance where human activity occurs, but may not be environmentally suitable or economically feasible where transmission is not focal, or in dynamic, high-flow environments where snails move across large areas. Other snail control options, such as biological snail control using natural predators, could overcome some of these challenges by providing widespread and continual snail population suppression, if predator density were maintained–via predator protection or augmentation–at an adequately high level.

Ultimately, better understanding the ecological drivers of schistosome transmission–for one or more species simultaneously–can help to identify and target high-risk environments for monitoring and control. This is especially important as global strategies to curb schistosomiasis transmission increasingly embrace environmental interventions to complement drug-based interventions [9]. Tailoring environmental interventions to the ecological and social settings where they are needed most may help bring us closer to the global goal of schistosomiasis elimination.

## Supporting information

S1 Appendix Additional information on methods. Detailed information on field sampling and object-based image analysis for classifying remotely-sensed satellite and drone imagery.
(DOCX)

S1 Fig Inclusion criteria for data used in statistical analyses. Schematic adapted from [20] showing which villages and water contact sites were included in each of our analyses. Manual snail surveys (left, Analysis 1) were conducted at all 16 villages beginning in May 2016. A subset of the 16 villages were involved in a parallel manipulative experiment that began in Spring 2017. Therefore, Analysis 1 (assessing snail–habitat analyses) excludes site–time combinations that were experimentally manipulated. Deep water snail sampling (Analysis 2) was conducted by boat in July–August 2017 in a subset of the 16 unmanipulated villages. Assessment of human infection outcomes (Analysis 3) given remote-sensed ecological and morphological water access site features required high-resolution or drone imagery available up to 120 m from water access site shorelines. Of the 16 study villages, only 12 villages met this criterion in year 1 (March 2016 to February 2017), and only 14 villages met this criterion in year 2 (March 2017 to February 2018).
(TIFF)

S2 Fig Human and environmental sampling timeline. Timeline adapted from [20] for drone deployment and snail sampling within water access sites (blue); snail sampling both inside water access sites and also in deep water and “offshore” areas outside water access sites (green); human schistosome screening (red); and administration of praziquantel to infected individuals (pink). Screen = urine and stool collection and filtration, PZQ = praziquantel administration. Tick marks delineate months.
(TIFF)

S3 Fig Snail–habitat associations. Results of analysis of snail–habitat associations within water access sites. Both intermediate host snails (*Bulinus* spp. on the left, and *Biomphalaria pfeifferi* on the right) were found at their highest densities in non-emergent vegetation at villages located on a river setting (top panels). This trend was also observed for *Bulinus* spp. snails at villages on a lake setting, but *Biomphalaria pfeifferi* snails were found in equal densities within non-emergent and emergent vegetation at villages on a lake setting (bottom panels). *p < 0.05, **p < 0.01, ***p < 0.001.
(TIFF)

S1 Table Village-level infection prevalence and egg burden. Infection prevalence and egg burden (egg intensity) for all participants in the longitudinal cohort study at baseline in 2016, and re-infection in 2017 and 2018 following treatment. Egg intensity is the geometric mean egg count for all positive infections (>1 egg/10ml urine or >1 egg/g feces), and % heavy infections is the percent of all participants that are considered to harbor high intensity infections (>50 eggs/ml urine for *S*. *haematobium* and >200 eggs/g feces for *S*. *mansoni*).
(XLSX)

S2 Table Infection presence models for *S*. *haematobium*. Infection presence (binomial GLMM) model results for *S*. *haematobium*, comparing models that integrated non-emergent vegetation coverage in nearshore extents (1 m to 5 m sampling radii) and offshore extents (45 m to 120 m sampling radii). All predictors were centered and scaled. Bolded p-values represent statistically significant factors.
(XLSX)

S3 Table Egg burden models for *S*. *haematobium*. Egg burden (negative binomial GLMM) model results for *S*. *haematobium*, comparing models that integrated non-emergent vegetation coverage in nearshore extents (1 m to 5 m sampling radii) and offshore extents (45 m to 120 m sampling radii). All predictors were centered and scaled. Bolded p-values represent statistically significant factors.
(XLSX)

S4 Table Infection presence models for *S*. *mansoni*. Infection presence (binomial GLMM) model results for *S*. *mansoni*, comparing models that integrated non-emergent vegetation coverage in nearshore extents (1 m to 5 m sampling radii) and offshore extents (45 m to 120 m sampling radii). All predictors were centered and scaled. Bolded p-values represent statistically significant factors.
(XLSX)

S5 Table Egg burden models for *S*. *mansoni*. Egg burden (negative binomial GLMM) model results for *S*. *mansoni*, comparing models that integrated non-emergent vegetation coverage in nearshore extents (1 m to 5 m sampling radii) and offshore extents (45 m to 120 m sampling radii). All predictors were centered and scaled. Bolded p-values represent statistically significant factors.
(XLSX)

S6 Table Infection presence interaction models for *S*. *haematobium* vs *S*. *mansoni*. Results of infection presence models (binomial GLMM) testing for differences in infection outcomes in children for *S*. *haematobium* vs. *S*. *mansoni*, given water access site shoreline length, circumscription, and snail habitat (non-emergent vegetation). Differences in infection outcomes for the two species were assessed using an interaction term for each ecological variable of interest and schistosome species. Shown are models that integrated non-emergent vegetation coverage in nearshore extents (1 m to 5 m sampling radii) and offshore extents (45 m to 120 m sampling radii). All predictors were centered and scaled. Bolded p-values represent statistically significant factors.
(XLSX)

S7 Table Egg burden interaction models for *S*. *haematobium* vs *S*. *mansoni*. Results of egg burden models (negative binomial GLMM) testing for differences in infection outcomes in children for *S*. *haematobium* vs. *S*. *mansoni*, given water access site shoreline length, circumscription, and snail habitat (non-emergent vegetation). Differences in infection outcomes for the two species were assessed using an interaction term for each ecological variable of interest and schistosome species. Shown are models that integrated non-emergent vegetation coverage in nearshore extents (1 m to 5 m sampling radii) and offshore extents (45 m to 120 m sampling radii). All predictors were centered and scaled. Bolded p-values represent statistically significant factors.
(XLSX)
