# Supplementary material for: Schistosome infection in Senegal is associated with different spatial extents of risk and ecological drivers for Schistosoma haematobium and S. mansoni
Source: PLoS Negl Trop Dis. 2021 Sep 27;15(9):e0009712. doi: 10.1371/journal.pntd.0009712 (PMC8476036; doi:10.1371/journal.pntd.0009712)
Supplement: S1 Appendix — Detailed information on field sampling and object-based image analysis for classifying remotely-sensed satellite and drone imagery. (DOCX) [file pntd.0009712.s001.docx]

# S1 Appendix: Additional information on methods

1. *Snail sampling within water access sites*

For quadrat-level snail sampling within water access sites, we used exhaustive snail sampling techniques to estimate snail density in each of three microhabitat types that dominate water access sites: non-emergent vegetation (*Ceratophyllum* spp., *Ludwigia* spp., *Potamogeton* spp., others); mix of non-emergent and emergent vegetation (predominantly *Typha* spp. and *Phragmites* spp.); and open water/mud. At each water access site, *Bulinus* spp*.* and *Biomphalaria pfeifferi* snails were collected in 15 quadrats (76.2-cm length × 48.26-cm width × 48.26-cm height; area = 0.3677 m2). Quadrat sampling locations were randomly distributed, but the number of samples taken per microhabitat type at a given water access site was proportional to the percent cover of microhabitat within that water access site, as determined through classified satellite and drone imagery and ground-truthed by two independent field technicians. High-resolution satellite and drone-based mapping, image classification, and microhabitat quantification methods are described below and in Chamberlin *et al*. [1]. In addition to snail counts, we recorded water depth, all vegetation to family or genus, and non-emergent floating vegetation mass at each quadrat. Snails were labelled by quadrat ID and later screened for schistosome infection in the laboratory via live parasite shedding and snail dissection.

1. *Satellite- and drone-based vegetation mapping and quantification*

Beginning in January 2017, we introduced drones to field sampling. A DJI Phantom 4 quadcopter equipped with a 1/2.3-inch CMOS (complementary metal-oxide-semiconductor) sensor and 12.4-megapixel camera was used to map each water access site during each snail sampling period. To map the freshwater ecosystems within and outside water access sites, we flew the drone in an overlapping grid at a fixed elevation, assuring 60–80% image overlap. We later merged the images and generated a high-resolution, georeferenced orthomosaic geotiff file using the open-source software OpenDroneMap. Drone imagery data were then imported into the image analysis software eCognition (Trimble Inc., Sunnyvale, CA, USA), and we classified the imagery into land, open water/mud, non-emergent floating/submerged vegetation, and emergent vegetation (Fig 1d) using object-based image analysis (OBIA). Using this approach, pixels are first grouped together into “objects” based on a heterogeneity score derived from parameters of scale, shape, compactness, and weights assigned to each image band (red, green, blue) [2].

Before classification, each image was segmented into objects that were small enough in size to distinguish between the functional classes under study using the multiresolution segmentation algorithm in eCognition. We then visually labeled a subset of objects to use as training samples for machine learning (ML) classifiers. Several ML classifiers were tested (Bayes, SVM, Decision Tree, Random Trees, and KNN) and their respective effectiveness was assessed visually. For most images, the k-nearest neighbor (KNN) algorithm produced the most accurate results. Some image distortion occurred because of reflected sunlight and required manual correction of some objects using additional drone data and field sampling data as validation.

As the drone was not a part of our field protocols during the first two sampling periods in spring and summer 2016, we used high-resolution satellite imagery to measure snail habitat in 2016. DigitalGlobe Foundation (now Maxar Technologies, Westminster, CO) provided access to WorldView-2 (WV-2) imagery of our study sites from June 29, 2016 (15 lake sites) and July 4, 2016 (9 river sites), in between our 2016 spring and summer field sampling periods (study year 1). WV-2 multispectral (MS) imagery consists of 8 spectral bands at 2 m spatial resolution including near-infrared, which improves image classification accuracy between biological and physical surface features [3]. We pansharpened the MS imagery using the supplied 0.5 m resolution panchromatic data and ArcGIS Pro to generate 0.5 m resolution MS imagery, which was possible to visually interpret into the four classifications by the eCognition image analyst using the same methods described above for drone imagery.

1. *Measuring snail habitat within different sampling radii at water access sites*

Snail habitat (non-emergent vegetation) was quantified from high-resolution satellite and drone imagery obtained during the summer field season during each 12-month period, after which schistosome re-infection rates were measured in children. This timing follows regional peak snail population growth during April through August [4]. To quantify snail habitat within the various sampling radii of interest, we first used ArcGIS to digitize the shoreline of each water access site using drone imagery. We next used the digitized shorelines to make areas extending 1 m or 5 m from shore, to assess the association between the area of nearshore snail habitat (commonly the focus of routine snail monitoring for schistosomiasis [5]) and human infection risk. We then generated areas extending from the centroids of shorelines out to 45 m, 60 m, 75 m, 90 m, 105 m, or 120 m (overlapping disk buffers as shown in Fig 1d, Fig 4a). The maximum distance of 120 m was chosen because it encompasses the maximum distance from shorelines within which drone imagery was available to assess snail habitat density. We presumed this range of areas was reasonable based on (i) a previous modeling study, calibrated on data from southern Africa, that estimated that *Bulinus* spp. and *Biomphalaria* spp. schistosome infection prevalence are highest in the vicinity of ~60 m from major water access sites [6], and (ii) a previous field experiment in St. Lucia, which showed that in flowing water, *S. mansoni* cercariae could infect mice nearly 100 m downstream of its source [7]. Disc buffers of 15 m and 30 m were excluded because their diameters were unreasonably small (encompassing only small parts of the shoreline) for many water access sites. Next, using classified drone imagery, we quantified the total (cumulative) area of each microhabitat (non-emergent vegetation, emergent vegetation, and water/mud) within each buffer area for each study year.

1. *Characterizing water access site size and shape characteristics*

We hypothesized that size and shape characteristics of each water site might impact snail abundance, parasite transmission efficiency, and huma–water contact frequency. We suspected that site circumscription, or whether or not a discrete water access site was mostly or fully enclosed by emergent vegetation, might influence snail density. This could arise where site circumscription restricts snail population connectivity between snail habitat in enclosed water access sites and more extensive snail habitat in the main channel of the river or open expanses of the lake. Circumscription might also influence snail abundance and mobility by reducing water flow rates inside water access sites. Lastly, circumscription might influence larval parasite swimming efficiency by reducing water flow (however, larval schistosome density was not assessed here). We also suspected that the linear length of a water access site’s shoreline might influence human transmission risk. This could arise if water access site size influences human–water contact frequency (i.e., larger sites are used more frequently than smaller ones). Like circumscription, water access site size might also influence snail density and larval parasite transmission efficiency, potentially mediated by water flow. Water flow impacts could also be mediated by the width of water access site openings (the distance between emergent vegetation site “walls” where they meet the river channel or open lake water). The width of water access site openings could also influence connectivity between snails within water access sites and snail populations in non-emergent vegetation outside water access sites. Last, we suspected that the amount of edge habitat inside water access sites (linear length of site edges, or emergent vegetation walls, from shoreline to open water) could influence snail density. This is based on personal observations that non-emergent vegetation tends to accumulate along the emergent vegetation “walls” of water access sites, and because non-emergent vegetation has been previously associated with elevated density of *B. globosus/truncatus* snails and elevated burden of *S. haematobium* in humans [8].

To test these hypotheses, we used overhead drone imagery obtained in July–August 2018 to record village-level: (i) site circumscription; (ii) linear width of the water access site shoreline; (iii) linear length of edge habitat at each water access site; and, (iv) offshore linear width of water access site openings (where discrete water access sites meet the main river channel or open lake water). For villages with more than one water access site, village-level circumscription was determined as a weighted average of site-level binary circumscription designations, weighted by water access site size (water access site size = [(shoreline width + offshore opening width)/2]*length). This assumes that larger sites are used more frequently by humans. Other site characteristics were estimated as sums across all water access sites (i.e., village-level shoreline width is the sum of shoreline width at all major water access sites used by village residents).

1. *Quantifying snail habitat within shoreline buffers of increasing spatial extents*

Using ArcGIS Pro, we created a digitized shoreline for each water access site using the boundary between land and water-access area; shorelines extended until they reached the emergent vegetation that forms the edges of water access sites. We used the traced water access site shorelines to create buffer areas that extended from shorelines 1m and 5m into the aquatic areas of water access sites. These near-shore polygons represent typical snail survey areas, because they are accessible, safely sampled and are locations where transmission presumably occurs. From the shoreline midpoint, multiple circular, overlapping disk buffers were generated at 15 m increments up to 120 m (Fig 1d, Fig 4a). Where buffer zones spatially overlapped with adjacent water access sites, the overlapping areas were split and equally distributed between the two sites so that no buffer areas overlapped. We then overlaid the multiple disk buffers on the classified satellite and drone imagery obtained during July–August field campaigns and measured the exact amount of non-emergent vegetation integrated across the 1-m, 5-m, and each 15-m buffer distance. The timing at which we measured non-emergent vegetation corresponds to the peak transmission season for schistosomiasis in this region, where peak snail abundance and snail infection prevalence occurs mid-summer, following snail population growth during the spring and early summer [4].

References

1. Chamberlin AJ, Jones IJ, Lund AJ, Jouanard N, Riveau G, Ndione R, et al. Visualization of schistosomiasis snail habitats using light unmanned aerial vehicles. Geospat Health [Internet]. 2021 Jan 8 [cited 2021 Jun 24];15(2). Available from: https://geospatialhealth.net/index.php/gh/article/view/818

2. Baatz M, Schäpe A. Multiresolution Segmentation: an optimization approach for high quality multi-scale image segmentation. 2000;12.

3. DeFries RS, Townshend JRG. NDVI-derived land cover classifications at a global scale. International Journal of Remote Sensing. 1994 Nov 1;15(17):3567–86.

4. Sturrock RF, Diaw O-T, Talla I, Niang M, Piau J-P, Capron A. Seasonality in the transmission of schistosomiasis and in populations of its snail intermediate hosts in and around a sugar irrigation scheme at Richard Toll, Senegal. Parasitology. 2001 Nov;123(7):77–89.

5. WHO. Field use of molluscicides in schistosomiasis control programmes: an operational manuel for programme managers. Geneva, Switzerland: World Health Organization (WHO); 2017.

6. Woolhouse ME, Chandiwana SK. Spatial and temporal heterogeneity in the population dynamics of Bulinus globosus and Biomphalaria pfeifferi and in the epidemiology of their infection with schistosomes. Parasitology. 1989 Feb;98 ( Pt 1):21–34.

7. Upatham ES. Dispersion of St. Lucian Schistosoma mansoni cercariae in natural standing and running waters determined by cercaria counts and mouse exposure. Annals of Tropical Medicine & Parasitology [Internet]. 2016 Mar 15 [cited 2021 Mar 10]; Available from: https://www.tandfonline.com/doi/abs/10.1080/00034983.1974.11686957

8. Wood CL, Sokolow SH, Jones IJ, Chamberlin AJ, Lafferty KD, Kuris AM, et al. Precision mapping of snail habitat provides a powerful indicator of human schistosomiasis transmission. PNAS. 2019 Nov 12;116(46):23182–91.
