# Supplementary figures and images for: Schistosome infection in Senegal is associated with different spatial extents of risk and ecological drivers for Schistosoma haematobium and S. mansoni
Source: PLoS Negl Trop Dis. 2021 Sep 27;15(9):e0009712. doi: 10.1371/journal.pntd.0009712 (PMC8476036; doi:10.1371/journal.pntd.0009712)

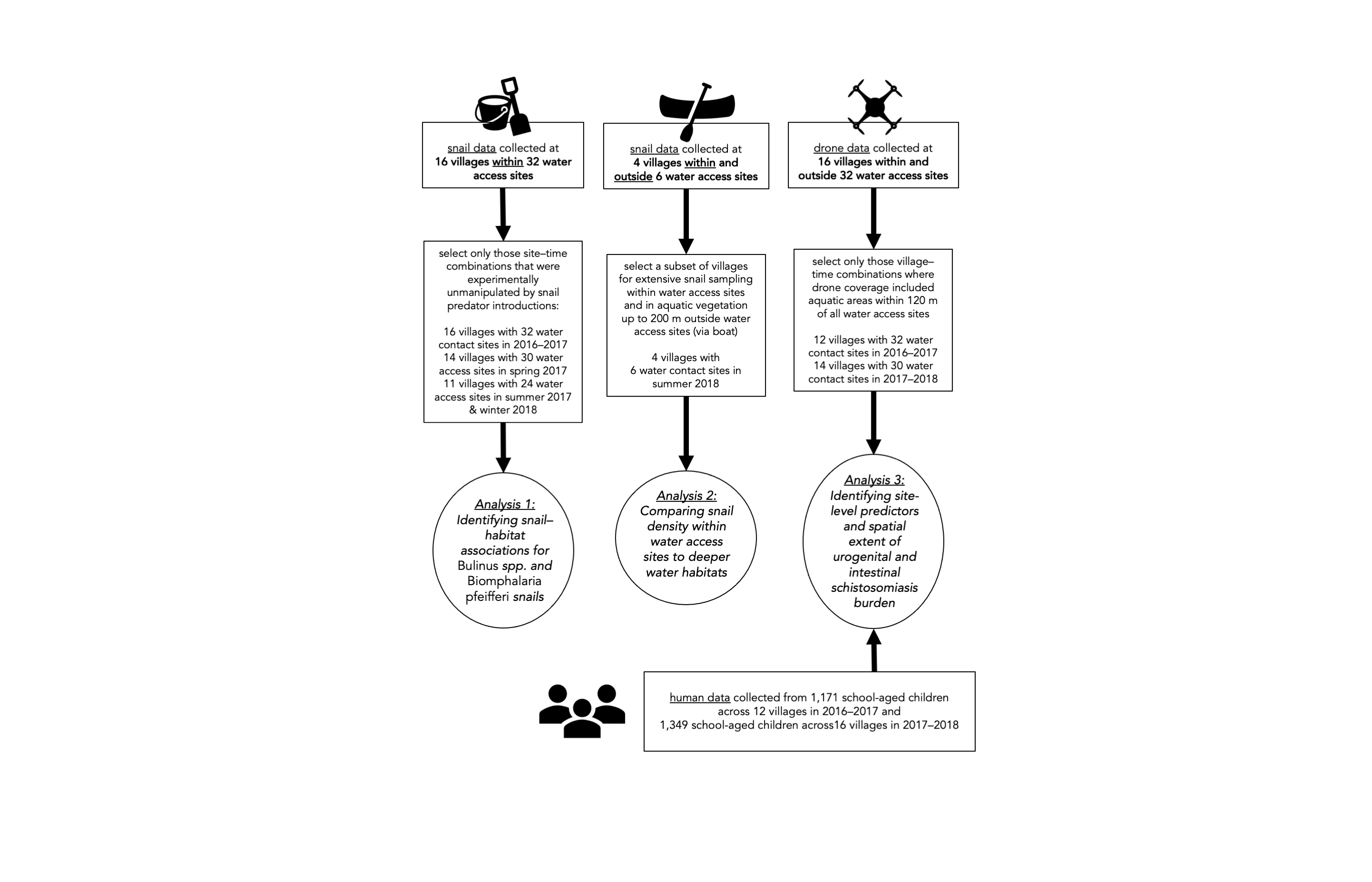

Supplement: S1 Fig — Schematic adapted from [20] showing which villages and water contact sites were included in each of our analyses. Manual snail surveys (left, Analysis 1) were conducted at all 16 villages beginning in May 2016. A subset of the 16 villages were involved in a parallel manipulative experiment that began in Spring 2017. Therefore, Analysis 1 (assessing snail–habitat analyses) excludes site–time combinations that were experimentally manipulated. Deep water snail sampling (Analysis 2) was conducted by boat in July–August 2017 in a subset of the 16 unmanipulated villages. Assessment of human infection outcomes (Analysis 3) given remote-sensed ecological and morphological water access site features required high-resolution or drone imagery available up to 120 m from water access site shorelines. Of the 16 study villages, only 12 villages met this criterion in year 1 (March 2016 to February 2017), and only 14 villages met this criterion in year 2 (March 2017 to February 2018). (TIFF) [file pntd.0009712.s002.tiff]

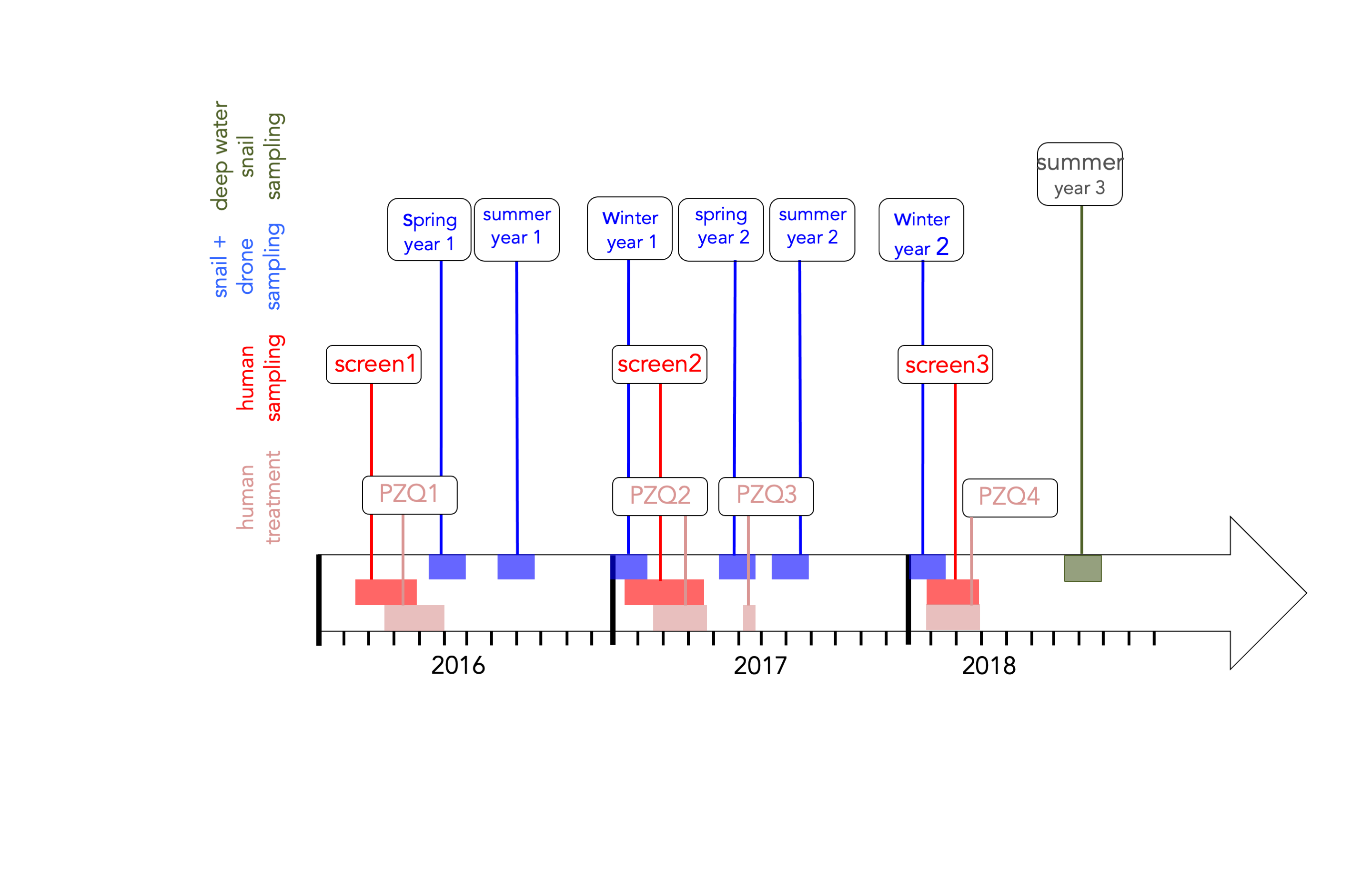

Supplement: S2 Fig — Timeline adapted from [20] for drone deployment and snail sampling within water access sites (blue); snail sampling both inside water access sites and also in deep water and “offshore” areas outside water access sites (green); human schistosome screening (red); and administration of praziquantel to infected individuals (pink). Screen = urine and stool collection and filtration, PZQ = praziquantel administration. Tick marks delineate months. (TIFF) [file pntd.0009712.s003.tiff]

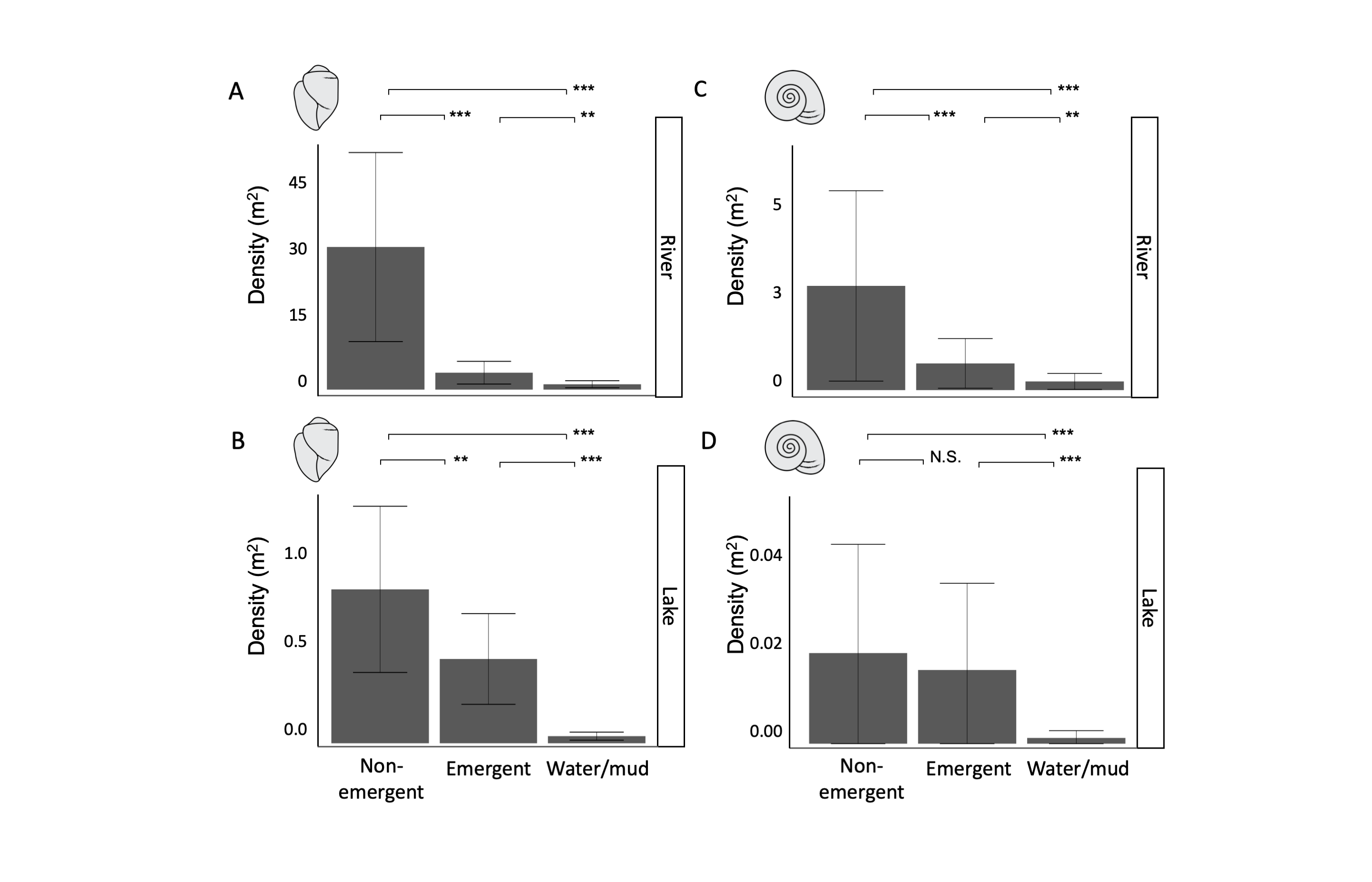

Supplement: S3 Fig — Results of analysis of snail–habitat associations within water access sites. Both intermediate host snails (Bulinus spp. on the left, and Biomphalaria pfeifferi on the right) were found at their highest densities in non-emergent vegetation at villages located on a river setting (top panels). This trend was also observed for Bulinus spp. snails at villages on a lake setting, but Biomphalaria pfeifferi snails were found in equal densities within non-emergent and emergent vegetation at villages on a lake setting (bottom panels). *p < 0.05, **p < 0.01, ***p < 0.001. (TIFF) [file pntd.0009712.s004.tiff]
